# Fecal and Environmental Shedding of Influenza A Virus in Brazilian Swine: Genomic Evidence of Recent Human-to-Swine Transmission

**DOI:** 10.3390/pathogens14080753

**Published:** 2025-07-31

**Authors:** Nágila Rocha Aguilar, Beatriz Senra Alvares da Silva Santos, Bruno Zinato Carraro, Brenda Monique Magalhães Rocha, Jardelina de Souza Todao Bernardino, Ana Luiza Soares Fraiha, Alex Ranieri Jeronimo Lima, Gabriela Ribeiro, Alessandra Silva Dias, Renata Rezende Carvalho, Bruna Ferreira Sampaio Ribeiro, Marta Giovanetti, Luiz Carlos Júnior Alcântara, Sandra Coccuzzo Sampaio, Maria Carolina Quartim Barbosa Elias Sabbaga, Rafael Romero Nicolino, Zélia Inês Portela Lobato, Maria Isabel Maldonado Coelho Guedes, Cesar Rossas Mota Filho, Vincent Louis Viala, Bruna Coelho Lopes, Erica Azevedo Costa

**Affiliations:** 1 Laboratório de Pesquisa em Virologia Animal, Escola de Veterinária, Universidade Federal de Minas Gerais, Belo Horizonte 31270-901, Brazil; naaguilar@hotmail.com (N.R.A.); beatrizsenra.santos@gmail.com (B.S.A.d.S.S.); brendammrocha@hotmail.com (B.M.M.R.); anasoaresfraiha@hotmail.com (A.L.S.F.); renitexx@gmail.com (R.R.C.); brunafsampaio.vet@gmail.com (B.F.S.R.); ziplobato@gmail.com (Z.I.P.L.); mariaisabel.guedes@gmail.com (M.I.M.C.G.); 2 Integrall Soluções em Produção Animal, Patos de Minas 38700-052, Brazil; bruno@integrall.org; 3 Centro para Vigilância Viral e Avaliação Sorológica (CeVIVAS), Instituto Butantan, São Paulo 05503-900, Brazil; jardelina.bernardino@fundacaobutantan.org.br (J.d.S.T.B.); alex.lima@fundacaobutantan.org.br (A.R.J.L.); gabriela.rribeiro@fundacaobutantan.org.br (G.R.); sandra.coccuzzo@butantan.gov.br (S.C.S.); carolina.eliassabbaga@butantan.gov.br (M.C.Q.B.E.S.); vincent.viala@butantan.gov.br (V.L.V.); 4 USDA-ARS-National Animal Disease Center Ames, 1920 Dayton Ave, Ames, IA 50010, USA; alessandrasilvadias@yahoo.com.br; 5 Mosquitos Vetores: Endossimbiontes e Interação Patógeno-Vetor, Instituto René Rachou-Fiocruz, Belo Horizonte 30190-002, Brazil; giovanetti.marta@gmail.com (M.G.); luiz.alcantara@fiocruz.br (L.C.J.A.); 6 Climate Amplified Diseases and Epidemics (CLIMADE), Belo Horizonte 30190-002, Brazil; 7 Sciences and Technologies for Sustainable Development and One Health, Università Campus Bio-Medico di Roma, 00128 Roma, Italy; 8 Laboratório de Bioestatística e Geoprocessamento, Escola de Veterinária, Universidade Federal de Minas Gerais, Belo Horizonte 31270-901, Brazil; rafael.nicolino@gmail.com; 9 Laboratório de Microbiologia, Departamento de Engenharia Sanitária e Ambiental, Escola de Engenharia, Universidade Federal de Minas Gerais, Belo Horizonte 31270-901, Brazil; cesar@desa.ufmg.br (C.R.M.F.); bruna.coelho.lopes@gmail.com (B.C.L.); 10 Laboratório de Bioquímica, Centro de Desenvolvimento Científico, Instituto Butantan, São Paulo 05503-900, Brazil

**Keywords:** influenza A virus, swine, fecal shedding, environmental surveillance, reverse zoonosis, pH1N1, H3N2, phylogenetics

## Abstract

Surveillance of swine influenza A virus (swIAV) traditionally focuses on respiratory matrices, yet emerging evidence suggests that fecal shedding and secondary environmental contamination may also contribute to viral dissemination. In this study, we collected and analyzed nasal, rectal, environmental, milk, and colostrum samples from naturally infected pigs in a commercial farm in Minas Gerais, Brazil. IAV RNA was detected in 25% of samples, including 42% from asymptomatic animals, with nasal swabs showing higher detection rates (30%) than rectal swabs (20%), though rectal Ct values were consistently higher, indicative of lower viral loads. We successfully isolated viable viruses from feces and effluent samples. Whole-genome sequencing revealed co-circulation of enzootic pH1N1 clade #2 (HA) and pN1 clade #4 (NA), alongside human-origin H3N2 sequences clustering within clade 3C.2a1b.2a.2a.1, and N2 segments related to pre-3C human lineages from 2001 to 2002. Phylogenetic and p-distance analyses support both recent reverse zoonosis and historical transmission events. Detection of complete HA/NA sequences from rectal swabs and treated effluent further emphasizes the surveillance value of non-respiratory matrices. The integration of respiratory and fecal/environmental sampling appears important to achieve more comprehensive IAV monitoring in swine herds and may have significant implications for One Health strategies in Brazil and beyond.

## 1. Introduction

Influenza A viruses (IAVs) are segmented, negative-sense, single-stranded RNA viruses of the Orthomyxoviridae family that affect a broad range of hosts, including birds, humans, and pigs. Pigs are considered “mixing vessels” due to the presence of both α2,3 and α2,6 sialic acid receptors in their respiratory tract, facilitating the replication and reassortment of avian and human IAV strains. Consequently, pigs play a key role in the ecology and evolution of IAVs, with significant implications for public and animal health [1,2].

The IAV genome comprises eight gene segments encoding at least ten proteins. Genetic reassortment between different lineages can lead to the emergence of novel strains with pandemic potential. Since the 2009 pandemic, the pH1N1 virus has become endemic in swine populations worldwide, co-circulating with other lineages such as classical H1N1, human-like H1 (huH1), and human-derived H3N2. In Brazil, following multiple introductions of human seasonal IAVs—including H1N1, H1N2, and H3N2—into swine populations, the pH1N1 virus has not only established endemic transmission but also undergone reassortment with previously circulating human-origin strains. These events led to the emergence of genetically diverse lineages and several reassortant viruses currently co-circulating in Brazilian swine herds, as highlighted in recent phylogenetic and phylodynamic studies [3,4].

Surveillance of IAVs in pigs is typically performed using nasal swabs due to the virus’s respiratory tropism. However, studies have reported the detection of IAV RNA in the feces of both humans and pigs, indicating potential intestinal shedding or environmental contamination. In swine, experimental infections with avian- and human-origin IAVs have demonstrated the presence of viral RNA in the intestinal tract and feces. This may be explained by the expression of both α2,3- and α2,6-linked sialic acid receptors on intestinal epithelial cells, suggesting the potential for local replication or viral persistence in this tissue [5,6]. In humans, the virus has been detected in feces for extended periods, even after respiratory shedding has declined, raising questions about similar shedding patterns in pigs [7,8,9].

During outbreaks, the presence and viability of swine-origin IAV (swIAV) in environmental matrices—such as airborne particles, oral fluids, and fomites—have been consistently demonstrated [10]. Environmental transmission has also been inferred from the early infection of suckling piglets through contaminated udder skin of lactating sows. In human public health, wastewater surveillance has emerged as a valuable strategy for monitoring IAV circulation, with viral RNA levels in sewage correlating with community incidence and supporting early outbreak detection [11]. In swine production, however, effluent sampling has typically been restricted to the detection of environmentally stable DNA viruses—such as circoviruses and adenoviruses—routinely excreted in feces [12]. The use of effluent for RNA virus detection, including IAV, remains underexplored in swine herds, despite its potential for environmental surveillance and understanding indirect transmission routes.

Wastewater-based surveillance has emerged as an important tool for monitoring virus circulation in populations, capable of detecting asymptomatic carriers and identifying emerging viral subtypes, including IAV and SARS-CoV-2 [11,12,13]. The adaptation of this approach to swine production systems could provide valuable insight into the dynamics of IAV circulation, especially when combined with fecal and environmental sampling.

Although fecal–oral transmission of IAVs has not been well documented in mammals, the detection of viable virus in feces and environmental samples raises concerns about its potential contribution to viral spread within herds and into the surrounding environment. Thus, implementing a practical and effective sampling system for feces and environmental matrices may enhance early detection and surveillance of IAV lineages circulating within swine herds. Understanding the role of fecal shedding and environmental contamination in IAV ecology is key to improving biosecurity strategies, mitigating the risk of viral persistence and spread within farms, and ultimately protecting both animal and public health.

In addition to characterizing IAV circulation through conventional sampling, this study also explores the potential of alternative matrices—including feces, colostrum, milk, and wastewater—for viral detection and genomic surveillance. These unconventional sources, although less frequently studied, may offer valuable insights into transmission dynamics and environmental persistence. Moreover, the study provides evidence of reverse zoonotic events involving human-origin H3N2 strains, highlighting the porous interface between human and swine IAV populations. By integrating molecular detection, viral isolation, and phylogenetic analysis, the findings contribute to a broader understanding of influenza ecology in swine production systems and reinforce the importance of a One Health approach in IAV monitoring strategies.

In this context, the present study aimed to (i) assess the detection rates and viral loads in nasal, rectal, and environmental samples; (ii) isolate viable viruses from feces and effluents; and (iii) perform phylogenetic and molecular characterization of the circulating IAV strains.

## 2. Materials and Methods

### 2.1. Ethics Approval

The study was approved by the Ethics Committee on Animal Use (CEUA) of the Universidade Federal de Minas Gerais, Brazil (protocol number 259/2023, approved on 16 August 2023).

### 2.2. Farm Description 

In 2023, a total of 603 samples were collected from a farrow-to-finish commercial swine farm located in Minas Gerais state, southeastern Brazil, with informed consent from the farmer owner. These included 265 nasal swabs and 266 rectal swabs from 266 individual animals, as well as 13 environmental samples and 59 milk and colostrum samples.

The farm houses approximately 70,000 pigs across various production phases. Over a four-year period, the farm experienced recurrent respiratory disease outbreaks, predominantly affecting piglets in the farrowing and nursery phases, with a mortality rate of approximately 10%. Other categories, such as growing and finishing pigs, were also affected intermittently.

The present study was conducted as a cross-sectional investigation, involving a single sampling of clinical and environmental materials. At the time of sampling, respiratory disease was actively affecting piglets starting from approximately 20 days of age in the farrowing phase and all age groups within the nursery phase. Piglets younger than 20 days did not exhibit clinical signs of respiratory disease. According to the farm veterinarian, respiratory signs typically emerged around 14 days of age and spread within cohorts in a cyclical manner, with weekly morbidity affecting 60–80% of piglets in a given batch. Clinical resolution was often followed by re-emergence of symptoms, suggesting sustained viral circulation within age groups. This cyclic dynamic, observed consistently across batches over several years, supports the interpretation of active transmission at the time of sampling. Although the proportion of affected animals decreased in recent years, viral circulation remains ongoing. Due to the high morbidity and clinical severity of the respiratory outbreaks, particularly among nursery pigs, animals presenting clinical signs were routinely treated with systemic antibiotics and anti-inflammatory drugs, as part of the farm’s standard therapeutic protocol. This approach aimed to reduce complications arising from opportunistic bacterial co-infections, including *Pasteurella multocida*, *Haemophilus parasuis*, and *Salmonella* spp., which were frequently detected during diagnostic investigations conducted by the farm's veterinarian.

To mitigate these outbreaks, the farm initially adopted mass vaccination using a commercial inactivated pH1N1 vaccine, administered to breeding sows and weaned piglets at four-month intervals over a ten-month period. Despite this strategy, influenza outbreaks persisted, prompting the adoption of an autogenous vaccine targeting pH1N1, H3N2, and huH1 viruses, administered to sows during the final third of gestation.

### 2.3. Water and Effluent Management System

The farm operates a multi-stage effluent treatment system. Effluent is first homogenized in a large collection tank, and the liquid fraction proceeds to a biodigester. For reuse purposes, the effluent from the receiving pond is treated in a biological reactor with aerators and then transferred to a settler. Settled solids are returned to the reactor, while the supernatant flows into a polishing pond and then into a settling pond. The latter supplies the Wastewater Treatment Plant for Water Reuse, which includes a flotation tank, a sludge removal tank, a treated water receiving tank, and chemical dosing units. The final treated water is stored in impermeable membrane-lined ponds and is pumped to distribution tanks used for gravity-fed washing of pig pen floors.

The drinking water supplied to pigs is sourced from an 80-meter-deep underground reservoir and stored in stainless steel tanks. No treatment is applied prior to distribution, as the groundwater is considered suitable for direct consumption.

### 2.4. Sampling Procedures

During the sampling period, clinical signs of respiratory disease—including coughing, sneezing, prostration, weight loss, and diarrhea—were observed in piglets aged approximately 20 days and older in the farrowing phase, as well as in all nursery pigs. These symptomatic animals were preferentially selected for sample collection. In the other categories, including replacement gilts, gestating sows, and pigs in the growing and finishing phases—where no clinical signs of respiratory disease were observed—simple random sampling was conducted.

Nasopharyngeal secretions were collected using 15 cm synthetic swabs, while rectal swabs were obtained by gently introducing the same type of swab into the rectum. Swabs were placed in 3 mL of phosphate-buffered saline (PBS) supplemented with 100 U/mL penicillin and 100 µg/mL streptomycin.

Environmental samples included 500 mL of raw waste from the shed outlet, reused water, and drinking water, collected in sterile containers, homogenized, stored in duplicates, and kept at −80 °C until analysis.

### 2.5. Milk and Colostrum Collection

A total of 59 samples were collected, comprising 17 colostrum and 42 milk samples. Samples were obtained after administering 10 IU of oxytocin intravenously into the auricular vein using a 20-gauge, 1.5-inch needle, as previously described [14].

In the first collection phase, 23 samples (7 colostrum and 16 milk) were collected without udder sanitization. In the second phase, 36 samples (10 colostrum and 26 milk) were collected following a rigorous udder sanitization protocol involving disposable wipes and 80% alcohol. Approximately 20 mL of each sample was manually collected from a pool of functional teats, homogenized, and stored in duplicate sterile containers at −80 °C.

### 2.6. Viral Detection and Subtyping

Viral RNA was extracted from nasal and rectal swabs using the QIAamp Viral RNA Mini Kit (QIAGEN, Hilden, Germany) and from environmental samples using the AllPrep DNA/RNA Micro Kit (QIAGEN, Hilden, Germany), according to the manufacturer’s instructions.

Universal detection of IAVs was performed using a one-step real-time reverse transcription PCR (RT-qPCR) targeting the conserved Matrix (M) gene, following the protocol described [15] with probe adaptation from [16]. Reactions were conducted using the GoTaq^®^ Enviro RT-qPCR System (Promega, Madison, WI, USA) in a final volume of 10 µL containing 5 µL of GoTaq^®^ Enviro Master Mix 2×, 0.2 µL of GoScript™ Enzyme Mix, 0.4 µM of each primer (AM-FW: 5′-GAGTCTTCTAACMGAGGTCGAAACGTA-3′; AM-RV: 5′-GGGCACGGTGAGCGTRAA-3′), and 0.25 µM of hydrolysis probe (AM-Probe: 5′-JUN-TCAGGCCCCCTCAAAGCCGAG-QSY-3′). Cycling conditions included 45 °C for 15 min (reverse transcription), 95 °C for 2 min, followed by 45 cycles of 95 °C for 15 s and 55 °C for 1 min. Amplification was performed on a QuantStudio™ 5 Real-Time PCR System (Applied Biosystems, Foster City, CA, USA). Reactions were performed in duplicate, and a non-template control was included in each run.

To determine the limit of detection (LoD), a standard curve was constructed using 10-fold serial dilutions (10^−1^ to 10^−14^) of a previously characterized Brazilian swine pH1N1 isolate (GenBank accession: JQ666849) obtained through virus isolation. Each dilution was tested in triplicate. The lowest dilution consistently amplified in all replicates corresponded to a Ct value of 39 and was adopted as the positivity threshold in this study. While the use of a virus isolate does not allow for absolute genome copy quantification, it enables reliable relative Ct-based estimation under field conditions. The *β-actin* gene was used as a housekeeping control to monitor RNA integrity and amplification efficiency, as described by [17].

HA subtyping was performed using nested RT-PCR assays with subtype-specific primers designed based on Brazilian swine IAV strains, as previously described by [18]. Separate reactions were carried out for pH1, huH1, and H3 subtypes, and the resulting amplicons were visualized by agarose gel electrophoresis. For NA subtyping (N1 and N2), specific singleplex RT-PCR protocols were employed for each target, as described by [19].

### 2.7. Virus Isolation

To assess viral viability, IAV-positive samples from nasal swabs, rectal swabs, and feces were diluted 1:3 in tenfold-concentrated PBS supplemented with 1% antibiotics (penicillin and streptomycin), and filtered through 0.22 µm membranes. A volume of 200 µL from each filtered sample was inoculated into 10- to 11-day-old specific-pathogen-free chicken embryonated eggs, incubated at 37 °C for 72 h. Allantoic fluid was then harvested [19].

Subsequent passages were conducted in MDCK (CCL-34) cells, which were maintained at 37 °C with 5% CO^2^ in complete Dulbecco’s Modified Eagle Medium (DMEM; SIGMA) containing 1 mM sodium pyruvate, 4.5 mg/mL L-glucose, 100 U/mL penicillin, 100 µg/mL streptomycin, and 5% fetal bovine serum [20].

### 2.8. Sequencing and Phylogenetic Analysis

Positive samples were subjected to full-genome amplification of IAV using multisegment reverse transcription PCR (MS-RT-PCR) with universal primers described by [21]. Briefly, 5 µL of RNA were used as template in a 25 µL one-step RT-PCR reaction with the SuperScript™ III One-Step RT-PCR System with Platinum™ Taq High Fidelity DNA Polymerase (Invitrogen, Carlsbad, CA, USA). The reaction included a mixture of influenza-specific universal primers targeting the conserved 12–13 nucleotide regions at the termini of all eight IAV genomic segments: Opti1-F1 (5′-GTTACGCGCCAGCAAAAGCAGG; 0.064 µM), Opti1-F2 (5′-GTTACGCGCCAGCGAAAGCAGG; 0.116 µM), and Opti1-R1 (5′-GTTACGCGCCAGTAGAAACAAGG; 0.176 µM). Thermal cycling conditions were performed as described by [22], optimized for efficient amplification of all segments. Amplified products were visualized by agarose gel electrophoresis, and successfully amplified samples were submitted for sequencing library preparation and next-generation sequencingaccording to the methodology outlined in [23], which used only clinical samples without prior isolation of the virus strain. In all sequencing runs, we included a high-titer isolated sample as a library prep positive control and water as a cross-contamination negative control. Raw reads were assembled using the VIPER pipeline (https://github.com/alex-ranieri/viper, accessed on 12 January 2025). Resulting segment assemblies were subsequently validated by read mapping with BBMap and then visually inspected and corrected if necessary, using Geneious software (2025.2). Contigs with coverage lower than 75% on segment coding sequences were discarded for phylogenetic analysis. Multiple sequence alignments were performed with MAFFT v7, and phylogenetic trees were inferred using the maximum likelihood (ML) method implemented in RAxML v8.2.11 through the MEGA12 platform. Bootstrap support was estimated using 1000 replicates. All trees were midpoint-rooted and visualized in the Interactive Tree of Life (iTOL v6), with nodes arranged in descending order of bootstrap support.

To contextualize the Brazilian swine IAV sequences, separate phylogenetic trees were constructed for each gene segment (H1, H3, N1, and N2), incorporating local sequences and representative references from key clades. Reference panels were selected to ensure coverage of (1) Brazilian swine-origin HA and NA sequences, previously classified [3,4], representing major clades (e.g., pH1 clades #1–4; pN1 clades #1–6; H3 clades 1990.5.1, 1990.5.2, 1990.5.3; N2 clades #1–6); (2) human H3N2 clades 3C.2a1b.2a.2a.1 and 3C.2a1b.2a.2b, circulating in Brazil in 2022–2023, retrieved from GISAID; (3) historic human H3 sequences from 1998, retrieved from GenBank, which shared the highest nucleotide identity with Brazilian swine H3 sequences based on BLASTn analysis; (4) swine H3 sequences from Chile, representative of clade 1990.5.4 and its ancestral clade 1900.5; (5) swine H3 clade 2010.1 reference sequences from U.S. swine [24]; (6) historic human N2 sequences, retrieved from GenBank, dated from 2001 to 2002, which shared the highest nucleotide identity with Brazilian swine N2 sequences based on BLASTn analysis.

A reduced set of 2–3 representative sequences per clade was selected based on year, geographic origin, and sequence completeness to ensure optimal tree resolution and clarity. The complete list of accession numbers and selection criteria is provided in Supplementary Table S1.

### 2.9. Statistical Methods

Data analysis was carried out using Microsoft Excel (Microsoft Corp., Redmond, WA, USA). Spreadsheets were created to systematically organize and tabulate the raw data across animal categories and age. Statistical descriptive analyses were employed, where the proportions of observed events were calculated by dividing the frequency of each event by the total number of animals in each category. This method provided a clear illustration of the relative distribution of events within the study population.

## 3. Results

### 3.1. Viral Detection in Nasal and Rectal Swabs

From the total of 531 samples (nasal and rectal swabs from individual pigs), 25% (132/532) were positive for IAV. Among the positive animals detected across all categories, 56% (61/110) were from pigs that did not exhibit clinical respiratory signs at the time of sampling, particularly among incoming gilts, gestating sows, and piglets aged 1 to 10 days in the farrowing phase. These animals were sampled to assess potential subclinical infections and obtain a broader picture of viral circulation on the farm.

In the farrowing phase, IAV RNA was detected in 5% (3/66) of nasal and/or rectal swabs collected from piglets aged 1 day, despite the absence of respiratory symptoms. Conversely, positivity increased to 25% (17/68) among 20-day-old piglets, coinciding with the onset of clinical signs. In the nursery phase, where respiratory disease was widespread, a high detection rate was also observed.

Samples were selected based on both clinical status and representation across production phases. In symptomatic categories (e.g., nursery and older farrowing piglets), animals were selected based on signs of influenza-like illness, whereas in asymptomatic groups (e.g., 1- to 10-day-old piglets, gilts, and gestating sows), sampling was randomized to detect potential subclinical infections.

Samples were selected based on both clinical status and representation across the full range of production phases. In symptomatic categories (e.g., nursery and farrowing piglets), animals were selected based on signs of influenza-like illness, whereas in asymptomatic groups (e.g., 1- to 10-day-old piglets, gilts, pregnant sows), sampling was randomized to detect potential subclinical infections. 

**Figure 1:**
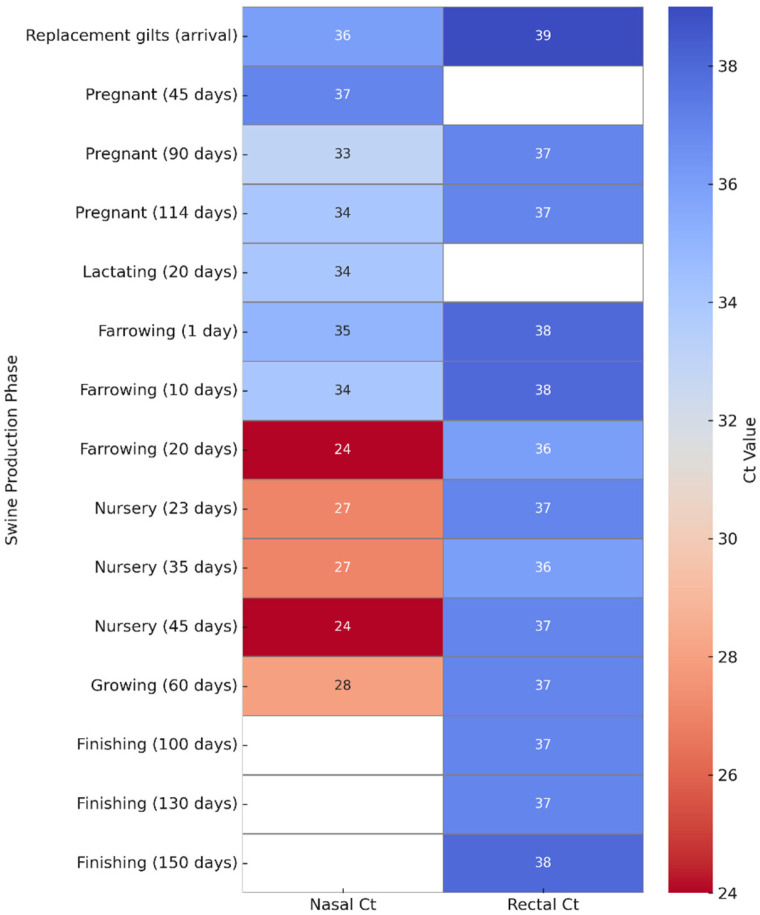
Heatmap of Influenza A virus Ct values in nasal and rectal swabs across swine production phases. Ct values were obtained by RT-qPCR from nasopharyngeal (left column) and rectal (right column) swabs collected at different production stages. Lower Ct values (indicative of higher viral load) are shown in dark red, while higher Ct values (suggestive of lower viral load or late infection) are represented in blue. Nasal swabs generally exhibited lower Ct values, particularly during the nursery phase (20–45 days of age), whereas rectal swabs showed higher Ct values across most categories, indicating lower viral loads. This visual comparison emphasizes the differential viral shedding patterns in respiratory versus enteric routes.

Using nasal swabs, IAV was detected at 30% (79/265) of samples, primarily among lactating sows, pregnant sows, nursery piglets, and farrowing piglets aged 20 days. Rectal swabs yielded a 20% (53/266) positivity rate, especially among nursery piglets and finishing pigs. Of the 132 positive samples, 17% (22) were simultaneously positive in both nasal and rectal swabs, particularly in nursery piglets and replacement gilts (Table 1). Among animals that tested positive exclusively in nasal swabs, 54% (31/57) were asymptomatic, including lactating or gestating sows and 1- to 10-day-old piglets, and 46% (26/57) exhibited respiratory symptoms. Among those positive exclusively in rectal swabs, 81% (25/31) were asymptomatic, while 19% (6/31), primarily nursery piglets and some older farrowing piglets, presented respiratory signs. Distinct shedding patterns were observed across production phases: Older animals in the finishing phase tested positive only via fecal samples, suggesting possible late-stage or residual fecal excretion, whereas lactating and gestating sows (e.g., at 45 or 114 days) were positive only in nasal swabs (Figure 1). These findings may reflect differential tissue tropism or temporal shedding patterns according to age and immune status.

The Ct values between nasal and rectal swabs depend on the production stage. Rectal swabs generally showed higher Ct values (36–40), with minimal variation between categories, suggesting low viral loads or intermittent fecal shedding.

For instance, rectal swabs from asymptomatic pigs had Ct values of 39 (incoming gilts), 37 (114 days gestation), and 38 (150-day finishing pigs).

In contrast, nasal swabs exhibited more variability. Marked reductions in Ct values were observed in the nursery phase (20–45 days of age), dropping to 24 in symptomatic piglets, reflecting active upper respiratory replication. In asymptomatic animals (e.g., sows in gestation or lactation), nasal swabs showed higher Ct values—37 at 45 days of gestation and 34 during lactation.

**Figure 2:**
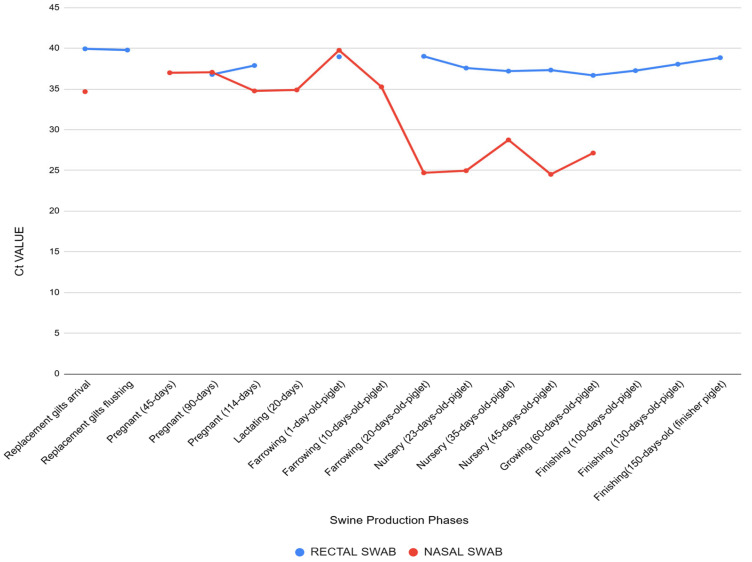
Cycle threshold (Ct) values obtained by RT-qPCR for IAV detection in nasal and rectal swabs collected from pigs at different stages of production. Each bar represents the mean Ct value observed in a specific production phase. Nasal samples showed lower Ct values during the nursery period (20–45 days of age), particularly in symptomatic piglets, indicating higher viral loads. In contrast, rectal samples exhibited consistently higher Ct values across all phases, including asymptomatic animals, suggesting lower levels of fecal viral shedding. These findings highlight differences in viral load dynamics between respiratory and enteric excretion routes.

This contrast is clearly illustrated in paired samples from the same animal. For example, in a 45-day-old nursery piglet, the nasal swab showed a Ct value of 24, while the corresponding rectal swab was 37, indicating higher viral replication in the respiratory tract compared to fecal excretion at this stage (Figure 2). These results underscore the complex dynamics of IAV shedding in swine and support the investigation of subtypes and environmental detection detailed in the following sections.

Among the nasal and rectal swab samples positive for IAV, a subset underwent subtyping to determine the circulating viral lineages. Subtyping was successful in 32 nasal swabs, revealing a predominance of H3 (41%), followed by pH1N1 (19%) and huH1 (13%). In rectal swabs, 34 samples were subtyped, with lower proportions of H3 (15%) and pH1N1 (18%); huH1 was not detected in fecal samples. These results suggest potential differences in viral lineage distribution or detection efficiency between respiratory and fecal routes.

**Figure 3:**
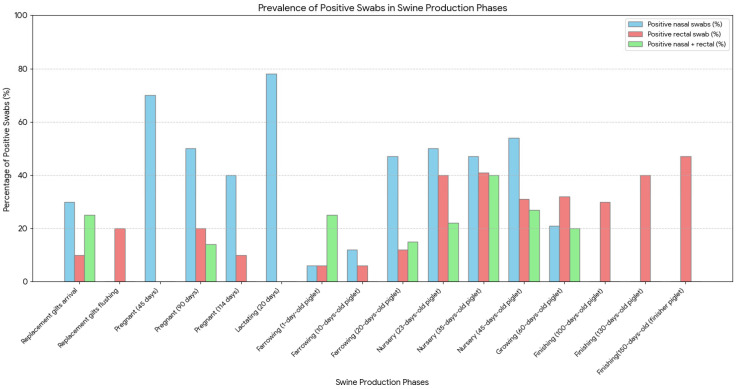
Frequency of Influenza A virus detection across swine production phases, by sample type. Blue bars represent animals positive by nasal swabs, red bars represent those positive by rectal swabs, and green bars represent animals testing positive in both swab types. Data are expressed as the percentage of positive swabs in each production category.

H3 subtype was more frequently detected in nursery piglets and sows, whereas pH1N1 appeared across multiple production stages. huH1 was identified exclusively in nasal swabs and milk samples, indicating its likely association with human-to-swine spillover events. A detailed summary of subtypes detected by sample type and production stage is provided in Table 2 and illustrated in Figure 3.

Regarding coinfection, 8% (5/66) of the subtyped samples showed dual infection, with 80% (4/5) positive for H3+H1hu and 20% (1/5) for pH1N1+H1hu. In addition, two animals tested positive in both nasal and rectal swabs collected at the same time point. Among these, one animal exhibited H3 in the nasal swab and a coinfection of pH1N1 and H3 in the rectal swab, and another tested positive for H3 in both sample types. These cases demonstrate both consistent and divergent subtype detection across anatomical compartments within individual animals.

Importantly, no piglet was simultaneously positive for H3 and huH1 in both nasal and rectal swabs at 10 days of age; the subtypes detected at that age were found in different individuals. To clarify the distribution of subtypes by production stage and sample type, a summary is presented in Table 2, with additional visualization in Figure 3.

These findings suggest that the detection of IAV RNA in rectal swabs may reflect prolonged fecal shedding or environmental contamination, rather than active replication in the intestinal tract. Such complexity in anatomical distribution reinforces the need for cautious interpretation of rectal swab positivity in surveillance studies.

### 3.2. Environmental Samples

A total of 13 environmental samples were collected, including raw waste from the shed outlet (*n* = 5), reclaimed reuse water (*n* = 6), and water from animal drinking troughs (*n* = 2). Of these, 4 samples (4/13; 31%) tested positive for IAV by RT-qPCR. Positive detections included two samples of reclaimed water, one from the finishing phase shed and another from the reservoir lagoon, as well as one raw waste sample from the maternity unit and one water sample from the drinking trough in the finishing area. These findings indicate the circulation of IAV genetic material in both effluent and drinking water systems within the farm environment.

Subtyping was performed on all positive environmental samples. One sample of reclaimed water from the reservoir lagoon tested positive for pH1N1, while another sample of reclaimed water used in the gestation barn was positive for huH1N1. Subtyping was not successful for the positive raw waste and drinking water samples, possibly due to low viral loads or RNA degradation in these matrices (Table 3).

These results underscore the importance of effluent-based surveillance strategies for IAVs. The detection of the huH1 subtype—a lineage of human origin—in reclaimed water also raises concern about reverse zoonotic events, supporting the hypothesis that human-origin viruses may not only infect pigs but persist in the shared farm environment. Such environmental persistence creates opportunities for inter-host transmission and viral reassortment, underscoring the need for integrated One Health approaches to monitor and mitigate influenza spread in intensive livestock settings.

### 3.3. Detection Rate of Milk and Colostrum Samples from Lactating Sows

Of the 59 milk and colostrum samples obtained from lactating sows in the maternity unit, 12 samples tested positive for IAVs, representing a detection rate of 20%. These positive samples were distributed among 3 colostrum samples (25%) and 9 milk samples (75%). Notably, 75% (9/12) of the positive samples were detected during the first collection—comprising 1 colostrum sample and 8 milk samples—while 25% (3/12) were identified during the second collection—comprising 2 colostrum and 1 milk sample.

All IAV-positive milk and colostrum samples originated from sows housed with piglets exhibiting respiratory symptoms. This association suggests that environmental contamination of the udder skin may contribute to viral presence in mammary secretions, particularly in the absence of strict hygiene protocols. Indeed, during the second collection phase, a rigorous udder sanitization procedure was implemented, coinciding with a noticeable reduction in positive samples. This supports the hypothesis that indirect contamination, rather than systemic excretion into milk, may account for the viral RNA detected.

Cycle threshold (Ct) values ranged from 37 to 39, indicating low viral loads consistent with superficial contamination or non-productive infection. Twelve milk and colostrum samples underwent subtyping, and among these, only one milk sample tested positive for the huH1 subtype. Although isolated, the detection of a human-origin IAV lineage in a lactating sow sample reinforces the need to consider the potential for reverse zoonosis, particularly in maternity sectors where close contact between humans and animals is frequent.

These findings underscore the importance of implementing and maintaining rigorous hygiene protocols in the maternity environment—not only to prevent pathogen dissemination to neonates but also to mitigate environmental contamination that may result in indirect routes of IAV transmission within swine herds.

### 3.4. Isolation of Positive Samples

Five samples were selected for virus isolation based on their lower Ct values, while also considering the need to represent different animal categories across the production system. The selected samples included three rectal swabs (from a sow at 90 days of gestation, a 35-day-old piglet, and a 130-day-old finishing pig), one nasal swab (from a 60-day-old piglet), and one environmental sample (effluent from the 150-day-old finishing barn) (Table 4). Each sample underwent six to seven passages in MDCK (CCL-34) cells to monitor the appearance of cytopathic effects (CPEs), such as cell rounding and detachment.

**Figure 4:**
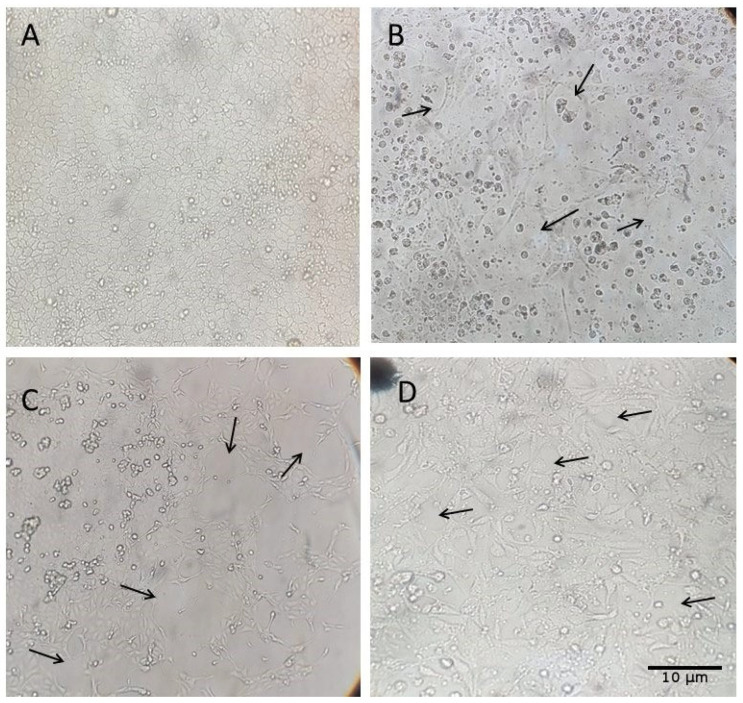
Cytopathic effects (CPEs) observed in MDCK (CCL-34) cell cultures after inoculation with Influenza A virus-positive samples from swine rectal swabs; MDCK CCL34 cells (Madin–Darby canine kidney). Black arrows highlight the cytopathic effects caused by influenza A, observed 72 h after inoculation. (**A**) Cell control inoculated with DMEM; (**B**) 7th inoculum of the rectal swab sample from a sow at 90 days of gestation; (**C**) 6th inoculum of the rectal swab sample from a 35-day-old piglet; (**D**) 6th inoculum of the rectal swab sample from a 130-day-old finishing pig. Magnification: 500×.

Among the rectal swab samples, CPE was first observed in the sample from the sow at 90 days of gestation at the fifth passage, reaching 60% by the seventh passage. In the sample from the 35-day-old piglet, CPE emerged at the fourth passage and increased to 80% by the sixth passage. The sample from the 130-day-old finishing pig showed faster progression, with CPE reaching 90% by the sixth passage (Figure 4).

**Figure 5:**
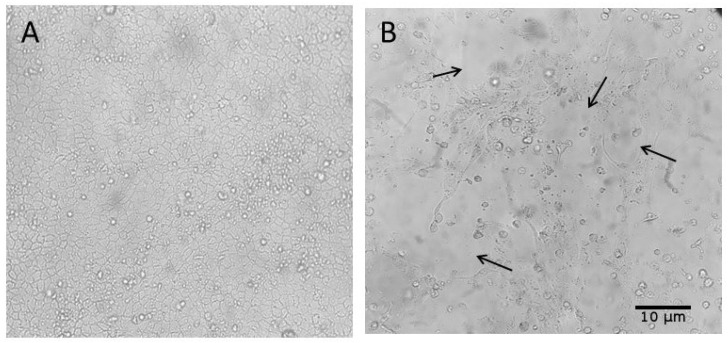
Cytopathic effects (CPEs) observed in MDCK (CCL-34) cell cultures after inoculation with Influenza A virus-positive samples from swine and environmental sources. Black arrows highlight the cytopathic effects caused by viral influenza infection. (**A**) Cell control inoculated with DMEM; (**B**) 6th inoculum of the effluent sample from the 150-day-old finishing barn. Magnification: 500×.

The effluent sample showed CPE beginning at the fourth passage, reaching 80% by the sixth passage (Figure 5). The nasal swab sample from the 60-day-old piglet exhibited pronounced CPE by the fifth passage, reaching 90% by passage six. RT-qPCR confirmed the presence of IAV in all inocula post-culture.

Among these five successfully isolated samples, two underwent subtyping. The nasal swab sample from the growing phase (60-days-old) was identified as H3N2, and the rectal swab sample from the 130-day-old pig was identified as pH1N1.

### 3.5. Sequencing and Phylogenetic Analysis

#### 3.5.1. Sample Selection and Sequencing Yield

For whole-genome sequencing (WGS) of the HA and NA segments of IAV, a total of 75 RT-qPCR-positive clinical samples were selected based on low Ct values. These included nasal swabs (*n* = 54), rectal swabs (*n* = 16), environmental samples (*n* = 4), and one inoculum sample obtained from viral isolation of a nasal swab. The samples originated from sows, piglets in maternity and nursery phases, and finishing pigs.

Due to the low quality of sequencing reads for internal gene segments, only the HA and NA gene sequences were analyzed. Among the 75 selected samples, 25 yielded paired HA and NA sequences, 5 yielded only HA sequences, and 7 yielded only NA sequences (Table 5).

#### 3.5.2. Subtype Classification

Among the 25 subtyped nucleotide sequences, 22 were identified as pH1N1 and 3 as H3N2. All 25 sequences were obtained from nasal swab samples. Of the five samples that yielded only HA sequences, four were classified as pH1Nx and one as H3Nx. Notably, two of the pH1Nx sequences originated from rectal swabs and three from nasal swabs, while the single H3Nx sequence was derived from a rectal swab. Among the seven samples that yielded only NA sequences, all were classified as HxN1; six were obtained from nasal swabs, and one from a maternity barn effluent sample (Table 5). Although the huH1 subtype (pre-pandemic human-origin H1) was detected by RT-qPCR subtyping in some clinical and environmental samples, no corresponding HA or NA sequences were recovered, likely due to low viral loads or RNA degradation.

#### 3.5.3. Host and Age Distribution

The nucleotide sequences obtained were predominantly recovered from nursery piglets during stages associated with overt respiratory symptoms. Specifically, among the 22 pH1N1 samples, all were nasal swabs collected from piglets aged 10 to 60 days, particularly at 20 and 45 days of age, when viral loads were highest. Similarly, the three H3N2 samples were derived from nasal swabs of piglets aged 20 and 35 days.

In the H1Nx group (*n* = 5), three sequences were obtained from 45-day-old piglets (one nasal swab and two rectal swabs), and two from 60-day-old piglets (nasal swabs). For the HxN1 group (*n* = 7), six sequences were recovered from nasal swabs of 45-day-old piglets, and one from a maternity barn effluent sample (Table 5). No full-length sequences corresponding to huH1 strains were recovered, despite their detection by RT-qPCR subtyping.

#### 3.5.4. Phylogenetic Characterization

**Figure 6:**
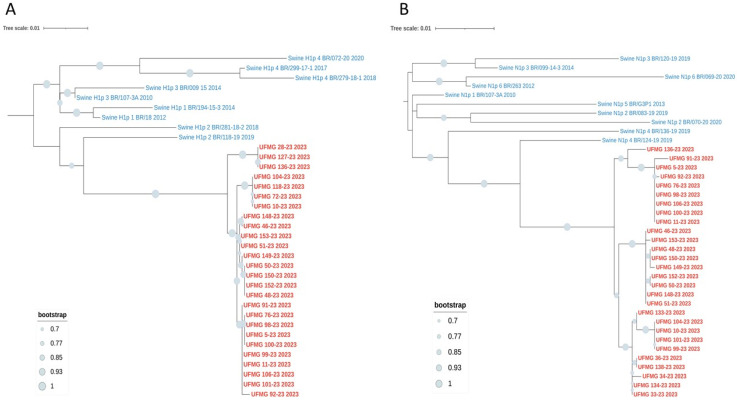
Phylogenetic analysis of HA and NA genes from pH1N1 strains detected in swine. (**A**) Maximum-likelihood phylogenetic tree of H1 hemagglutinin (HA) gene sequences. Red labels indicate sequences obtained in this study; blue labels represent reference swine pH1 sequences from clades #1–4. All UFMG sequences clustered within clade pH1-#2 with strong bootstrap support (≥70%) and low intra-clade nucleotide diversity (mean p-distance = 0.010). (**B**) Maximum-likelihood phylogenetic tree of N1 neuraminidase (NA) gene sequences. All N1 sequences from this study (in red) grouped within clade pN1-1, consistent with co-circulating pH1 clade #2 viruses. Reference Brazilian swine N1 sequences from clades #1–6 (in blue) were included. High bootstrap values (≥70%) and low intra-clade distance (mean p-distance = 0.009) confirmed close genetic relatedness. All trees were inferred using RAxML with 1000 bootstrap replicates, midpoint-rooted, and visualized using MEGA12.

Phylogenetic analyses based on the HA and NA gene segments revealed distinct lineage clustering of the IAV strains circulating in this study. All H1 sequences were classified within pH1 clade #2, sharing high nucleotide similarity and strong bootstrap support with previously described Brazilian swine pH1N1 strains from this clade (Figure 6A).

**Figure 7:**
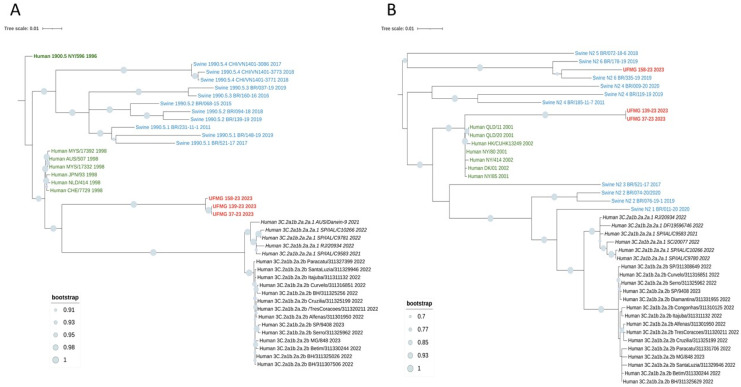
Phylogenetic analysis of HA and NA genes from H3N2 strains detected in swine. (**A**) Maximum-likelihood phylogenetic tree of H3 hemagglutinin (HA) gene sequences. Sequences obtained in this study (in red), including one from a rectal swab (UFMG_128), clustered within the human-like clade 3C.2a1b.2a.2a.1 (in italic black), showing high similarity to human H3N2 strains circulating in Brazil between 2021 and 2023. Reference sequences from swine clades 1990.5.1 to 1990.5.4 (in blue), historic human strains from 1998 (in green), and ancestral clade 1900.5 (dark green) were included for phylogenetic context. (**B**) Maximum-likelihood phylogenetic tree of N2 neuraminidase (NA) gene sequences. Two distinct clusters were observed among the three sequences from this study (in red): UFMG_158 grouped with Brazilian swine clade N2-#6, while UFMG_37 and UFMG_139 clustered with human-origin strains from 2001 to 2002 (pre-3C clade, in dark green), suggesting historical reverse zoonotic events. Reference sequences included swine N2 clades #1–6 (in blue) and contemporary human clades 3C.2a1b.2a.2a.1 and 3C.2a1b.2a.2b (in italic and regular black, respectively). All trees were inferred using RAxML with 1000 bootstrap replicates, midpoint-rooted, and visualized using MEGA12. Bootstrap values ≥ 70% are shown at major nodes.

The four H3 sequences (UFMG_37, UFMG_128, UFMG_139, and UFMG_158) clustered within the human-derived clade 3C.2a1b.2a.2a.1, which circulated in Brazil between 2021 and 2023. These swine sequences grouped tightly with recent human seasonal H3N2 strains, showing robust bootstrap support (≥95%), suggesting recent reverse zoonotic transmission from humans to pigs (Figure 7A). Although UFMG_128 (rectal swab) was excluded from the phylogenetic tree due to its shorter sequence length (1373 bp), pairwise comparison and clade-specific marker analysis confirmed its classification within clade 3C.2a1b.2a.2a.1, consistent with the other H3 sequences from this study. No sequences clustered within clade 3C.2a1b.2a.2b, reinforcing the specificity of this lineage association.

This phylogenetic placement was further supported by p-distance analysis, which revealed a mean pairwise nucleotide divergence of 0.016 between the swine H3 sequences and the human clade 3C.2a1b.2a.2a.1 sequences. In contrast, divergence was higher when compared to clade 3C.2a1b.2a.2b (0.037) and swine H3 clade 1990.5.2 (0.081) (Table 6).

Regarding the NA segments, the six N1 sequences were classified within pN1 clade #4, matching the co-circulating pH1 clade #2 viruses and showing high similarity to previously identified Brazilian strains (Figure 6B). In contrast, two of the three N2 sequences (UFMG_139 and UFMG_37) grouped with human seasonal viruses from 2001 to 2002, predating the emergence of modern 3C clades. These sequences did not cluster with enzootic swine N2 clades #4 or #6, strongly suggesting a historical human origin. The third N2 sequence (UFMG_158) fell within swine clade #6, consistent with endemic reassortants in Brazil. The phylogenetic placement of UFMG_139 and UFMG_37 was supported by p-distance analysis, which showed low divergence from 2001 to 2002 human N2 strains (0.022) but substantially higher divergence from swine clades #4 (0.071) and #6 (0.085). These findings point to reverse zoonotic events involving human-derived N2 segments, now persisting in the swine population. The reintroduction and maintenance of such lineages raise concerns about the potential for reassortment with endemic strains, underscoring the importance of sustained genomic surveillance at the human–animal interface.

Together, these findings highlight the continued circulation of endemic pH1N1 lineages in swine and provide strong molecular evidence for recent human-to-swine transmission events involving seasonal H3N2 viruses.

Among the sequences included in the phylogenetic analysis, two HA sequences classified as pH1Nx and one H3Nx were derived from rectal swabs, and one NA sequence classified as HxN1 was obtained from maternity barn effluent. These findings reinforce the relevance of non-respiratory matrices in genomic surveillance and support the detection of phylogenetically informative IAV genomes from fecal and environmental sources.

## 4. Discussion

This study confirms that IAV RNA can be detected in swine feces under natural conditions, aligning with prior experimental data [5,7]. Notably, 56% of positive animals were asymptomatic, with multiple categories—including pregnant and lactating sows, and finishing pigs—testing positive in both nasal and rectal swabs. These findings highlight the relevance of including asymptomatic individuals in surveillance programs, as they may contribute to sustained viral circulation on farms.

To contextualize our sampling strategy, it is important to note that our decision to select samples from various animal categories, including some without overt clinical signs, was intended to provide a broader view of viral circulation throughout the production system. This approach was based on the observation of persistent outbreaks across multiple age groups and phases, suggesting widespread and possibly asymptomatic transmission.

Although nasal swabs yielded higher detection rates (30%) than rectal swabs (20%), Ct values were markedly higher in the latter (mean 37), reflecting lower viral loads. This supports the hypothesis that IAV replicates more efficiently in the respiratory tract. Still, sustained detection of viral RNA in feces—especially from asymptomatic animals—suggests intermittent or prolonged shedding via the gastrointestinal route. In humans, respiratory shedding peaks within four days, whereas viral RNA in feces can persist for over three weeks [25,26,27], reinforcing this interpretation. Although studies on fecal shedding of Influenza A virus remain scarce, data from other respiratory RNA viruses support the plausibility of gastrointestinal excretion. Notably, SARS-CoV-2 RNA is frequently detected in feces for significantly longer periods than in respiratory samples. The median duration of fecal shedding ranges from 11 to 43 days, with reports of elimination lasting up to 71 days even after respiratory swabs tested negative and symptoms had resolved [28,29,30,31,32]. Although these findings are based on human and SARS-CoV-2 studies, they support the biological plausibility of prolonged fecal shedding of respiratory viruses in other hosts, such as pigs, especially in the context of low-level viral replication or delayed clearance.

Ct values also varied by production phase and clinical status. Symptomatic piglets in the nursery phase (20- to 45-days-old) exhibited the lowest Ct values (~24), indicating active viral replication. In contrast, asymptomatic gestating and lactating sows showed higher values (e.g., 37 at 45 days of gestation, 34 during lactation), or undetectable levels in some finishing pigs, reinforcing the correlation between nasal shedding and clinical signs. Notably, among the 87 pigs that presented respiratory symptoms at the time of sampling, only 56% tested positive for IAV RNA in at least one swab type. Several factors may explain this partial detection rate, including the timing of sampling in relation to the disease course. According to farm records and previous guidelines [33], optimal viral detection occurs during the febrile stage, which tends to peak in the early nursery phase. In addition, factors such as sample storage, handling, and transport may have influenced RNA stability and detection sensitivity. Furthermore, although the Influenza A virus was the primary target of this investigation, other respiratory pathogens were also present on the farm. Opportunistic bacterial infections, such as those caused by *Pasteurella multocida*, *Haemophilus parasuis*, and *Salmonella* spp., were frequently detected and may have contributed to clinical signs, especially in immunologically naive piglets or those experiencing co-infection. As for viral agents, porcine circovirus type 2 (PCV2) was a potential differential diagnosis; however, the farm maintained an effective PCV2 vaccination protocol, and no clinical or epidemiological evidence of PCV2-associated disease was observed at the time of sampling. Importantly, pigs exhibiting clinical respiratory signs but testing negative for IAV were not excluded from the study, allowing for a comprehensive assessment of the outbreak dynamics.

The isolation of viable virus from feces reinforces the potential for fecal–oral transmission or environmental contamination. While replication in enterocytes remains uncertain, the presence of α2,3- and α2,6-linked sialic acid receptors in the porcine intestinal tract supports the possibility of local infection [6]. Alternatively, swallowed virus-laden mucus may resist degradation due to protective mucins [26], allowing viral RNA to persist in feces. The presence of IAV antigens in immune cells such as monocytes, neutrophils, and macrophages [33,34,35,36] may contribute to viral dissemination and persistence. While productive replication in these cells remains debated [36,37], circulating monocytes may facilitate systemic dissemination of viral RNA due to their migratory capacity. In contrast, resident macrophages may support local persistence in tissues, potentially contributing to prolonged shedding.

Detection of IAV RNA in effluent samples from asymptomatic 150-day-old pigs, with 47% positivity in feces despite negative nasal swabs, emphasizes the importance of environmental surveillance. Virus isolation from these samples confirmed viability. In the maternity barn, piglets of varying ages tested positive in both swab types, and lactating sows exhibited 78% nasal positivity. These findings indicate that pigs shed IAV through both nasal and fecal routes, contributing to environmental contamination. Detection of viral RNA in reclaimed water after biological treatment further supports this risk. The persistence of viral RNA in treated effluent suggests incomplete removal by standard biological treatments, a pattern also observed for SARS-CoV-2 [38,39]. Although viral loads were lower in reclaimed water, their presence points to residual contamination and reinforces the value of wastewater surveillance.

The importance of RNA virus surveillance in swine wastewater has become increasingly evident since the COVID-19 pandemic, which demonstrated the feasibility of detecting and monitoring respiratory RNA viruses through wastewater-based epidemiology (WBE) [40]. Historically, DNA viruses were prioritized for environmental surveillance due to their greater stability; however, advancements in molecular detection techniques have enabled the recovery of RNA fragments even from complex environmental matrices. This approach is particularly relevant for IAV, an RNA virus with potential for rapid mutation and reassortment, whose environmental detection could enhance early warning systems, support outbreak tracking, and inform biosecurity practices in swine production systems.

It is important to note that the swab and effluent samples selected for virus isolation were not subtyped prior to inoculation. Given the concurrent circulation of multiple IAV subtypes within the herd, it is possible that some of the samples harbored mixed infections. During serial passaging in MDCK cells, the most replication-competent viral subtype may have outcompeted others, leading to preferential amplification and detection of a single strain. This potential bias should be considered when interpreting subtype identification following in vitro propagation and highlights the need for direct subtyping from primary material in future studies.

Detection of IAV RNA in milk and colostrum (20% positivity) from asymptomatic sows raises concerns about potential early-life exposure of piglets. Although only pH1N1 was identified among these samples, subtyping was limited due to high Ct values in most cases. The source of viral RNA in milk and colostrum remains uncertain; however, evidence from both experimental and field studies supports the hypothesis of environmental contamination of the mammary gland, particularly via respiratory secretions from infected piglets. For instance, ref. [41] demonstrated that up to 76% of udder wipe samples collected from nurse sows were positive for IAV RNA and that these sows contributed to transmission events during early suckling. In addition, ref. [42] experimentally detected IAV RNA in milk from infected ferrets, and similar findings have been reported in humans. Although vertical transmission through milk cannot be entirely ruled out, these findings underscore the role of close piglet-to-sow contact and environmental contamination in IAV dissemination. The integration of udder wipe sampling and colostrum/milk testing may help clarify these transmission pathways in future surveillance studies.

Phylogenetic analysis revealed the co-circulation of distinct IAV lineages within the studied herd. All H1 sequences were classified within pH1 clade #2, a lineage derived from the 2009 pandemic H1N1 virus. This clade was introduced into pigs during 2009–2011 and has since become endemic in Brazilian swine [4]. The N1 segments belonged to clade pN1 clade #4, a reassortant lineage also of pandemic origin. Although both clades descend from the same ancestral pH1N1 virus, their independent evolutionary trajectories and frequent reassortment have been documented in Brazil [3,4], supporting the genotypic constellation identified in our samples.

In contrast, the H3N2 sequences presented strong molecular evidence of recent reverse zoonosis. All three H3 sequences clustered within clade 3C.2a1b.2a.2a.1, a human seasonal lineage that circulated widely in Brazil between 2021 and 2023. This clade has been responsible for major human H3N2 outbreaks in multiple Brazilian states, including Minas Gerais, where the studied farm is located [23]. The detection of this clade in pigs, with high bootstrap support and low p-distance values relative to human strains, indicates a recent introduction from humans to swine.

Interestingly, the N2 sequences grouped with H3N2 human seasonal viruses from 2001 to 2002, genetically related to strains such as A/Moscow/10/99-like and A/Fujian/411/2002-like, which circulated globally before the emergence of clade 3C. These N2 genes likely represent historical introductions of human-origin viruses into pigs, previously unreported in Brazil. One of our swine sequences (UFMG_128) clustered closely with human N2 strains from 2002, reinforcing this inference.

Similar events involving human-derived H3 and N2 segments have recently been reported in Mexico and the southern United States [43,44], but to our knowledge, this is the first documented case in Brazil of reverse zoonotic transmission of clade 3C.2a1b.2a.2a.1 into pigs. These findings highlight the increasing frequency and global distribution of such events and the critical role of local surveillance in early detection.

Importantly, phylogenetically informative sequences were also recovered from non-respiratory samples. Among the HA sequences, two pH1Nx and one H3Nx originated from rectal swabs, while one NA sequence (HxpN1) was derived from maternity barn effluent. These results reinforce the value of environmental matrices—often overlooked in surveillance—for recovering complete or partial viral genomes. The successful detection and sequencing of these samples may be explained by contamination via respiratory secretions, but also by the expression of both α2,3- and α2,6-linked sialic acid receptors in the swine intestinal tract, which could support intestinal replication or persistence of the virus [6].

Importantly, the swine herd investigated in this study experienced a severe respiratory outbreak, characterized by intense clinical signs and an estimated mortality rate of 10%. Despite the use of autogenous vaccines, the outbreak persisted, raising concerns regarding vaccine efficacy. The genomic detection of human-origin H3N2 viruses—specifically from clade 3C.2a1b.2a.2a.1—in affected pigs suggests that the herd was likely immunologically naïve to this novel strain. This mismatch between the vaccine formulation and the circulating viruses may have contributed to the increased disease severity observed. These findings underscore the potential consequences of reverse zoonotic events in commercial herds and highlight the urgent need for continuous genomic surveillance and timely updating of swine vaccine strains.

Furthermore, the detection of human-origin H3N2 IAV in pigs, although uncommon in Brazil since the 1990s [3], deserves emphasis due to the inherent risk of genetic reassortment. The co-circulation of human and endemic swine IAV lineages provides an opportunity for gene segment exchange, potentially generating novel reassortant strains with unpredictable phenotypic properties. Recent studies in North and South America have documented increasing occurrences of reverse zoonoses involving seasonal H3N2 viruses, some of which resulted in reassortment with endemic swine viruses [43]. These events highlight the dynamic interface between human and swine IAV populations and the importance of early detection to mitigate public and animal health risks.

Together, our results demonstrate the co-circulation of enzootic pH1N1 viruses, human-origin H3N2 strains, and historically introduced N2 genes in a single herd, reflecting the complex evolutionary dynamics and reassortment potential of IAVs in swine. These findings underscore the importance of integrating fecal and environmental sampling into IAV monitoring strategies, as such approaches enhance detection sensitivity, facilitate early recognition of emerging reassortants, and provide a broader picture of viral circulation within production systems.

Some limitations regarding the subtyping of IAV-positive samples should be considered. Among the five samples submitted for virus isolation, only two could be successfully subtyped. One likely reason is that the subtype-specific RT-qPCR primers used for HA detection (targeting pH1N1, huH1, and H3) were specifically designed based on Brazilian strains [18], potentially reducing sensitivity for genetically divergent strains. Furthermore, the initial screening assay for IAV RNA (targeting the matrix gene) is more sensitive than the subtype-specific assays, which may fail to detect samples with lower viral loads or mismatches in primer regions. These challenges highlight the need for constant updating of molecular assays to match the evolving genetic diversity of circulating strains.

Additional methodological limitations may have affected the sensitivity of RT-qPCR in complex matrices such as milk, colostrum, and wastewater. These sample types often contain substances that inhibit PCR, including lipids, proteins, and organic debris. Viral RNA is also more prone to degradation in these matrices, especially during transport and storage. Environmental conditions such as temperature, pH, and enzymatic activity in wastewater can accelerate RNA decay. In the case of colostrum and milk, the high immunoglobulin content may interfere with RNA extraction or amplification efficiency. Although internal controls were included to assess reaction quality, the presence of high Ct values or undetermined subtypes in some samples may reflect low viral titers, RNA fragmentation, or suboptimal primer annealing. These factors should be taken into account when interpreting negative or inconclusive results and underscore the importance of assay optimization and the potential value of metagenomic approaches in future investigations.

Finally, in light of the emergence of HPAI H5N1 in Brazil, the role of pigs as potential intermediary hosts must be emphasized. A One Health framework, integrating molecular, clinical, and environmental surveillance, is essential to detect emerging IAV threats and prevent future pandemics.

## 5. Conclusions

This study provides novel evidence that Influenza A virus (IAV) RNA, including viable virus, can be detected in swine feces and effluent under natural conditions. These findings underscore the relevance of fecal and environmental matrices in IAV surveillance and highlight the potential for alternative transmission routes beyond the respiratory tract. The identification of IAV RNA in fecal samples from asymptomatic animals and the successful isolation of infectious virus suggest a possible role for gastrointestinal shedding in the persistence and spread of IAV within pig herds and into the environment.

Furthermore, the detection of human-origin H3N2 viruses (clade 3C.2a1b.2a.2a.1) in Brazilian pigs—combined with N2 segments related to pre-3C human strains from the early 2000s—provides strong molecular evidence of reverse zoonotic events and historical reassortments. The co-circulation of enzootic pH1N1 strains (clade #2) with reassorted NA segments (pN1 clade #4 and human-derived N2) within a single herd highlights the ongoing genetic evolution of swine IAVs in Brazil.

These results reinforce the need to integrate respiratory, fecal, and environmental sampling into routine surveillance programs. Such strategies will enhance early detection, improve our understanding of IAV ecology in swine populations, and help mitigate the zoonotic risks posed by emerging strains. A comprehensive One Health approach remains essential for anticipating and preventing future public and animal health threats.

## Figures and Tables

**Table 1:** Number and proportion of pigs testing positive for IAV RNA in nasal swabs, rectal swabs, or both sample types across production phases. Coinfection refers to pigs testing positive in both nasal and rectal swabs at the same sampling time. The proportion was calculated in relation to the total number of pigs tested per category. Animal categories highlighted in gray presented clinical respiratory signs at the time of sampling.

Swine Production Phases	Positive Nasal Swabs	Positive Rectal Swab	Positive Nasal + Rectal
Replacement gilts arrival	3/10 (30%)	1/10 (10%)	1/4 (25%)
Replacement gilts flushing	0/10 (0%)	2/10 (20%)	0/2 (0%)
Pregnant (45 days)	7/10 (70%)	0/10 (0%)	0/7 (0%)
Pregnant (90 days)	5/10 (50%)	2/10 (20%)	1/7 (14%)
Pregnant (114 days)	4/10 (40%)	1/10 (10%)	0/5 (0%)
Lactating (20 days)	7/9 (78%)	0/9 (0%)	0/7 (0%)
Farrowing (1-day-old piglet)	2/33 (6%)	2/33 (6%)	1/4 (25%)
Farrowing (10-day-old piglet)	4/33 (12%)	2/33 (6%)	0/9 (0%)
Farrowing (20-day-old piglet)	16/34 (47%)	4/34 (12%)	3/20 (15%)
Nursery (23-day-old piglet)	5/10 (50%)	4/10 (40%)	2/9 (22%)
Nursery (35-day-old piglet)	8/17 (47%)	7/17 (41%)	6/15 (40%)
Nursery (45-day-old piglet)	14/26 (54%)	8/26 (31%)	6/22 (27%)
Growing (60-day-old piglet)	4/19 (21%)	6/19 (32%)	2/10 (20%)
Finishing (100-day-old piglet)	0/10 (0%)	3/10 (30%)	0/4 (0%)
Finishing (130-day-old piglet)	0/10 (0%)	4/10 (40%)	0/4 (0%)
Finishing (150-day-old finisher piglet)	0/14 (0%)	7/15 (47%)	0/7 (0%)
Total	79/265 (30%)	53/266 (20%)	22/136 (16%)

**Table 2:** Distribution of Influenza A virus subtypes (pH1N1, H3N2, and huH1) detected by RT-PCR across swine production phases. Values represent the number of positive samples over the total tested for each subtype. The last column lists cases of coinfection in which nasal and rectal swabs collected from the same animal were positive, with corresponding subtype information.

Swine Production Phases	pH1N1	H3N2	huH1	Coinfections (Nasal and Rectal)
Replacement gilts arrival	0/4 (0%)	0/4 (0%)	0/4 (0%)	None
Replacement gilts flushing	0/2 (0%)	0/2 (0%)	0/2 (0%)	None
Pregnant (45 days)	0/0 (0%)	0/0 (0%)	0/0 (0%)	None
Pregnant (90 days)	0/4 (0%)	0/4 (0%)	0/4 (0%)	None
Pregnant (114 days)	2/4 (50%)	0/4 (0%)	0/4 (0%)	None
Farrowing (2-day-old)	1/2 (50%)	0/2(0%)	0/2 (0%)	None
Farrowing (1-day-old piglet)	0/3 (0%)	0/3 (0%)	0/3 (0%)	None
Farrowing (10-day-old piglet)	0/4 (0%)	1/4 (25%)	1/4 (25%)	None
Farrowing (20-day-old piglet)	0/16 (0%)	8/16 (50%)	1/16 (6%)	H3N2 (nasal and rectal swabs), *n* = 1
Nursery (23-day-old piglet)	1/6 (17%)	4/6 (67%)	0/6 (0%)	H3N2 (nasal swab), pH1N1+H3N2 (rectal swab), *n* = 1
Nursery (35-day-old piglet)	1/6 (17%)	1/6 (17%)	0/6 (0%)	None
Nursery (45-day-old piglet)	0/5 (0%)	2/5 (40%)	2/5 (40%)	None
Growing (60-day-old piglet)	0/4 (0%)	2/4 (50%)	0/4 (0%)	None
Finishing (100-day-old piglet)	0/2 (0%)	0/2 (0%)	0/2 (0%)	None
Finishing (130-day-old piglet)	0/2 (0%)	0/2 (0%)	0/2 (0%)	None
Finishing (150-day-old finisher piglet)	1/2 (50%)	0/2 (0%)	0/2 (0%)	None
Total	6	18	4	2

**Table 3:** Detection of Influenza A virus by RT-qPCR in environmental samples collected from different locations of a swine farm. The table indicates the sample type, location of collection, cycle threshold (Ct) values for positive samples, and viral subtype detected by RT-PCR. Among the 13 samples analyzed, 4 tested positive for IAV, including two from reclaimed water, one from raw effluent, and one from drinking water. The pH values of each sample are also presented, with mildly alkaline conditions observed in the reclaimed water samples, which may favor viral persistence.

Sample Type	Collection Site Building	Cycle Threshold (Ct)	Subtype Detected	pH
Reuse water	Ponds serving as reservoirs for reused water	36.758	pH1N1	7.8
Reuse water	Gestation building	36.589	huH1N1	7.9
Reuse water	Nursery building	Undetermined		8
Drinking water	Whole-farm storage tank	Undetermined		-
Drinking water	Farrowing waterer/watering system	Undetermined		7.1
Raw effluent	Replacement gilts building	Undetermined		-
Raw effluent	150-day finishing building	31.17	Not subtyped	7.6
Raw effluent	Post-chemical treatment slaughterhouse	Undetermined		6.5
Raw effluent	Nursery building	Undetermined		7.04
Raw effluent	Farrowing building	Undetermined		7.27
Raw effluent	Farrowing building	Undetermined		7.34
Raw effluent	Farrowing building	36.467	Not subtyped	7.71
Raw effluent	Farrowing building	Undetermined		7.69

**Table 4:** Influenza A virus-positive samples selected for virus isolation. Samples included nasal and rectal swabs and environmental effluent, selected based on lower Ct values and animal category.

Sample Type	Collection Site Building	Cycle Threshold (Ct)	Subtype Detected
Effluent	150-day finishing	31.17	Not performed
Rectal swab	Pregnant 90 days	36.00	Not performed
Rectal swab	Piglet 35 days	33.72	Not performed
Rectal swab	Finishing 130 days	32.66	pH1N1
Nasal swab	Growing 60 days	21.58	H3N2

**Table 5:** swIAV strains sequenced in this study with corresponding sample type, subtypes, and HA and NA sequences.

Strains	Sample Type	Subtype	HA Clade	HA CDS Cov. (%)	HA_GenBank Accession Number	NA Clade	NA CDS Cov. (%)	NA_GenBank Accession Number
A/swine/Brazil/10	Nasal swab	pH1N1	H1#2	100	PV876006	N1#4	97.9	PV876028
A/swine/Brazil/11	Nasal swab	pH1N1	H1#2	100	PV875989	N1#4	99.7	PV876018
A/swine/Brazil/46	Nasal swab	pH1N1	H1#2	100	PV875997	N1#4	97.9	PV876039
A/swine/Brazil/48	Nasal swab	pH1N1	H1#2	100	PV875999	N1#4	97.7	PV876045
A/swine/Brazil/50	Nasal swab	pH1N1	H1#2	100	PV876000	N1#4	97.9	PV876041
A/swine/Brazil/51	Nasal swab	pH1N1	H1#2	100	PV876002	N1#4	100	PV876016
A/swine/Brazil/76	Nasal swab	pH1N1	H1#2	100	PV875990	N1#4	99.4	PV876026
A/swine/Brazil/91	Nasal swab	pH1N1	H1#2	100	PV875991	N1#4	99.3	PV876025
A/swine/Brazil/92	Nasal swab	pH1N1	H1#2	100	PV875995	N1#4	99.6	PV876024
A/swine/Brazil/98	Nasal swab	pH1N1	H1#2	100	PV875996	N1#4	99.7	PV876019
A/swine/Brazil/99	Nasal swab	pH1N1	H1#2	100	PV875992	N1#4	92.7	PV876029
A/swine/Brazil/100	Nasal swab	pH1N1	H1#2	100	PV875994	N1#4	98.6	PV876022
A/swine/Brazil/101	Nasal swab	pH1N1	H1#2	100	PV876005	N1#4	93.1	PV876030
A/swine/Brazil/104	Nasal swab	pH1N1	H1#2	100	PV876007	N1#4	97.8	PV876031
A/swine/Brazil/106	Nasal swab	pH1N1	H1#2	100	PV875993	N1#4	100	PV876023
A/swine/Brazil/136	Nasal swab	pH1N1	H1#2	100	PV876008	N1#4	99.6	PV876035
A/swine/Brazil/148	Nasal swab	pH1N1	H1#2	100	PV875998	N1#4	97.9	PV876040
A/swine/Brazil/149	Nasal swab	pH1N1	H1#2	100	PV876004	N1#4	97.9	PV876044
A/swine/Brazil/150	Nasal swab	pH1N1	H1#2	100	PV876009	N1#4	97.8	PV876046
A/swine/Brazil/152	Nasal swab	pH1N1	H1#2	100	PV876001	N1#4	97.9	PV876042
A/swine/Brazil/153	Nasal swab	pH1N1	H1#2	100	PV876003	N1#4	100	PV876043
A/swine/Brazil/37	Nasal swab	H3N2	H3#2	100	PV876064	N2#6	100	PV876066
A/swine/Brazil/139	Nasal swab	H3N2	H3#2	100	PV876065	N2#6	100	PV876033
A/swine/Brazil/158	Nasal swab	H3N2	H3#2	100	PV876063	N2#6	100	PV876067
A/swine/Brazil/5	Nasal swab	pH1Nx	H1#2	100	PV876010	N1#4	99.5	PV876021
A/swine/Brazil/28	Nasal swab	pH1Nx	H1#2	100	PV876013	-	-	-
A/swine/Brazil/72	Rectal swab	pH1Nx	H1#2	100	PV876011	-	-	-
A/swine/Brazil/118	Nasal swab	pH1Nx	H1#2	100	PV876012	-	-	-
A/swine/Brazil/127	Nasal swab	pH1Nx	H1#2	100	PV876014	-	-	-
A/swine/Brazil/128	Rectal swab	H3Nx	H3#2	78.6	PV876062	-	-	-
A/swine/Brazil/33	Nasal swab	HxN1	-	-	-	N1#4	97.6	PV876036
A/swine/Brazil/34	Nasal swab	HxN1	-	-	-	N1#4	93.1	PV876037
A/swine/Brazil/36	Nasal swab	HxN1	-	-	-	N1#4	99.1	PV876038
A/swine/Brazil/55	Maternity barn effluent	HxN1	-	-	-	N1#4	38.9	PV876017
A/swine/Brazil/133	Nasal swab	HxN1	-	-	-	N1#4	97.5	PV876032
A/swine/Brazil/134	Nasal swab	HxN1	-	-	-	N1#4	99.6	PV876034
A/swine/Brazil/138	Nasal swab	HxN1	-	-	-	N1#4	99.6	PV876020

**Table 6:** Distribution of UFMG Influenza A virus samples according to segment and phylogenetic clade. Clade classification was based on maximum-likelihood analysis of the HA and NA genes using reference sequences from Brazilian swine, human-origin H3N2 clades circulating in Brazil (2021–2023), and historical human strains.

Segment	Clade	No. of UFMG Samples
H1	pH1-#2 (swine-like)	26
H3	3C.2a1b.2a.2a.1 (human-like)	4
N1	pN1-#4	28
N2	N2-#6 (swine-like)	1
N2	Pre-3C (human-like)	2

## Data Availability

All genome sequences generated in this study have been deposited in GenBank under accession numbers PV875989 to PV876014; PV876016 to PV876046; PV876062 to PV876068. Additional datasets analyzed during this study are included in the Supplementary Materials.

## Supplementary Materials

The following supporting information can be downloaded at https://www.mdpi.com/article/10.3390/pathogens14080753/s1, Table S1: Representative sequences used in the phylogenetic analysis of the H1, H3, N1, and N2 genes.
