# Supplementary material for: Fecal and Environmental Shedding of Influenza A Virus in Brazilian Swine: Genomic Evidence of Recent Human-to-Swine Transmission
Source: Pathogens. 2025 Jul 31;14(8):753. doi: 10.3390/pathogens14080753 (PMC12388998; doi:10.3390/pathogens14080753)
Supplement: Supplementary file 1 [file pathogens-14-00753-s001.zip › pathogens-3772411-supplementary.pdf]

Supplementary Material

Supplementary Table S1. Representative sequences used in the phylogenetic analysis of the H1, H3, N1, and N2 genes. Sequences were selected based on the following criteria: (i) year of collection to capture historical and recent diversity, (ii) host species (swine or human), (iii) geographic origin, with preference for Brazilian and reference international strains, and (iv) availability of complete or nearly complete sequences for each gene segment. These references supported the clade classification of Brazilian swine Influenza A virus sequences presented in the phylogenetic trees (Figure 6A–D).

ID Reference Sequences	Host/Country/Strain	Year
	**H1**	
**Clade #1**	**Brazilian Clade**	
KM496987*	Swine/BR/18	2012
MT041093*	Swine/BR/194-15-3	2014
**Clade #2**	**Brazilian Clade**	
MW772635*	Swine/BR/118-19	2019
MT041109*	Swine/BR/281-18-2	2018
**Clade #3**	**Brazilian Clade**	
KF683614*	Swine/BR/107-3A	2010
MH559923*	Swine/BR/009-15	2015
**Clade #4**	**Brazilian Clade**	
MT068715*	Swine/BR/299-17-1	2017
MW772603*	Swine/BR/072-20	2020
MT041053*	Swine/BR/279-18-1	2018
	**N1**	
**Clade #1**	**Brazilian Clade**	
KF683616*	Swine/BR/107-3A	2010
**Clade #2**	**Brazilian Clade**	
MW772803*	Swine/BR/070-20	2020
MW772860*	Swine/BR/083-19	2019
**Clade #3**	**Brazilian Clade**	
MH560013*	Swine/BR/099-14-3	2014
MW772645*	Swine/BR/120-19	2019
**Clade #4**	**Brazilian Clade**	
MW772518*	Swine/BR/124-19	2019
MW772677*	Swine/BR/136-19	2019
**Clade #5**	**Brazilian Clade**	
KP027603*	Swine/BR/G3P1	2013
**Clade #6**	**Brazilian Clade**	
KM497005*	Swine/BR/263	2012
MW772589*	Swine/BR/069-20	2020
	**H3**	
**Clade 1990.5.1**	**Brazilian Clade**	
KM507535*	Swine/BR/231-11-1/2011	2011
MT068745*	Swine/BR/521-17/2017	2017
MW772683*	Swine/BR/148-19/2019	2019
**Clade 1990.5.2**	**Brazilian Clade**	
MH559843*	Swine/BR/068-15/2015	2015
MT041197*	Swine/BR/094-18/2018	2018
MW772900*	Swine/BR/139-19/2019	2019

**Clade 1990.5.3**	**Brazilian Clade**	
MT068652*	Swine/BR/160-16/2016	2016
MW772770*	Swine/BR/037-19/2019	2019
**Clade 1990.5.4**	**Chilean Clade**	
MN054199*	Swine/CHI/VN1401-3771	2018
MN054344*	Swine/CHI/VN1401-3773	2018
MH347124*	Swine/CHI/VN1401-3086	2017
**Clade 1900.5**	**Human ancestor**	
CY010060*	Human/USA/596/1996 (NY)	1996
**Historical human origin (1997–1999)**		
CY112893*	Human_NLD/414	1998
AF382318*	Human/CHE/7729	1998
CY118618*	Human_MYS/17392	1998
CY118610*	Human/MYS/17332	1998
AB019356*	Human_JPN/93	1998
KM821319*	Human/AUS/507	1998
**Clade 3C.2a1b.2a.2b**	**Brazilian samples/Minas Gerais State**	
EPI ISL 18810142**	Human/BH/311307506	2022
EPI ISL 18810186**	Human/Betim/311330244	2022
EPI ISL 18810119**	Human/Alfenas/311301950	2022
EPI ISL 18810182**	Human/Santa Luzia/311329946	2022
EPI ISL 18810181**	Human/Paracatu/311327399	2022
EPI ISL 18810176**	Human/Serro/311325962	2022
EPI ISL 18810173**	Human/BH/311325256	2022
EPI ISL 18810172**	Human/Cruzilia/311325199	2022
EPI ISL 18810171**	Human/BH/311325026	2022
EPI ISL 18090803**	Human/MG/848	2023
EPI ISL 18810164**	Human/TresCoracoes/311320211	2022
EPI ISL 18810161**	Human/Curvelo/311316851	2022
EPI ISL 17806265**	Human/SP/9408/2023	2023
EPI ISL 18810152**	Human/Itajuba/311311132	2022
**Clade 3C.2a1b.2a.2a.1**	**Brazilian Strains**	
EPI ISL 13755350**	Human/SP/IAL/C9583/2021	2021
EPI ISL 14986698**	Human/SP/IAL/C9781/2022	2022
EPI ISL 16199725**	Human/SP/IAL/C10266/2022	2022
EPI ISL 15426896**	Human/RJ/20934/2022	2022
	**Reference Strain**	
EPI-ISL-197575**	Human/AUS/Darwin-9	2021
**N2**		
**Clade #1**	**Brazilian Clade**	
MW772533*	Swine/BR/011-20	2020
**Clade #2**	**Brazilian Clade**	
MW772486*	Swine/BR/074-20	2020
MW772823*	Swine/BR/076-19-1	2019
**Clade #3**	**Brazilian Clade**	
MT068747*	Swine/BR/521-17	2017
**Clade #4**	**Brazilian Clade**	
KM507521*	Swine/BR/185-11-7	2011

MW772502*	Swine/BR/119-19	2019
MW772752*	Swine/BR/009-20	2020
**Clade #5**	**Brazilian Clade**	
MT068669*	Swine/BR/072-18-6	2018
**Clade #6**	**Brazilian Clade**	
MW772912*	Swine/BR/178-19	2019
MW772709	Swine/BR/335-19	2019
**Pre 3C Clade (2000 e 2002)**		
CY003178*	Human/A/New York/414	2002
CY000387*	Human/New York/85	2001
CY000571*	Human/New York/80	2001
CY017525*	Human/QLD/20	2001
CY017485*	Human/QLD/11	2001
EU103825*	Human/DK/01	2002
EU857138*	Human/HK/CUHK13249	2002

*Sequences originating from GenBank; ** Sequences originating from GISAID
